# Malignant melanoma of the vagina: A case report and review of the literature

**DOI:** 10.3892/ol.2014.2357

**Published:** 2014-07-17

**Authors:** LIFENG CHEN, YIN XIONG, HUAN WANG, LIZHI LIANG, HUILING SHANG, XIAOJIAN YAN

**Affiliations:** 1 Department of Gynecology, Zhejiang Provincial People’s Hospital, Hangzhou, Zhejiang 310014, P.R. China; 2 Department of Gynecology, Cancer Center, Sun Yat-Sen University, Guangzhou, Guangdong 510060, P.R. China; 3 Department of Gynecology, The Third Affiliated Hospital of Sun Yat-Sen University, Guangzhou, Guangdong 510630, P.R. China; 4 Department of Gynecology, The First People’s Hospital of Foshan, Guangzhou, Guangdong 528000, P.R. China; 5 Department of Gynecology, The First Affiliated Hospital of Wenzhou Medical University, Wenzhou, Zhejiang 325000, P.R. China

**Keywords:** malignant melanoma, vagina

## Abstract

Primary malignant melanoma of the vagina is an extremely rare variant of melanoma that accounts for <3% of all vaginal malignancies. Primary malignant melanoma of the vagina has a worse prognosis as compared with non-genital melanomas or other vaginal malignant neoplasms. A-35-year-old female had a diagnosis of primary malignant melanoma of the vagina. A local excision of the tumor was first performed, followed by a radical excision as a further therapeutic measure. The patient returned after three weeks, presenting with a vesico-vaginal fistula. A conservative operation was subsequently performed in order to improve the quality of life of the patient. Pelvic metastases were identified 6 months after the completion of the last surgical therapy and subsequent follow-up examinations were performed in another hospital. The present case study describes the clinical features and surgical procedures of this patient with primary malignant melanoma of the vagina. In conclusion, melanoma of the vagina is an extremely aggressive cancer and the overall prognosis is poor despite the various treatment options.

## Introduction

Malignant melanoma is considered to arise from melanocytic cells in the skin and mucosal membranes. Malignant melanomas predominantly develop in areas that are exposed to sunlight, including the back or legs. Although it was first reported in 1887, there have been no more than 500 primary vaginal melanoma cases reported worldwide. Malignant melanomas always present as deeply invasive tumors, and the overall incidence of malignant melanoma among women is 0.46 per million (1). Malignant melanoma accounts for <10% of all female genital tract melanomas, for 2.4–2.8% of all vaginal malignancies, and for 0.3–0.8% of all malignant melanomas (2). Primary malignant melanoma of the vagina usually occurs in women aged in their 60s or 70s, with the majority of patients being postmenopausal (3,4). Patients commonly complain of vaginal bleeding, vaginal discharge or a palpable mass. The present study describes a case of vaginal melanoma and a discussion of the treatment options.

## Case report

A 35-year-old female, gravida 3, para 2, was admitted to the Gynecological Ward of The First People’s Hospital of Foshan (Guangzhou, China) with a chief complaint of vaginal bleeding following intercourse for 1 month. The father of the patient had a history of nasopharyngeal carcinoma. Clinical examination identified an ulcerated, mobile, and solid-cystic 4 cm-diameter mass on the left lateral aspect of the upper half of the vagina, with no indication of abnormality in the external genitalia, uterus or ovaries. There were no palpable lymph nodes. The patient was clinically diagnosed with a cyst of vagina, and thus a tumor resection of vaginal wall was performed. During the operation, the tumor presented as poorly circumscribed, gray-white, and easily bled at contact, resulting in difficulty to remove the entire tumor. Tissue samples were collected and sent for frozen-section examination, and a primary vaginal malignant tumor was reported, which accorded with the initial experience-based speculation of the surgeon. Following the final diagnosis shown through results from paraffin-sections, the patient and her family were fully informed about the disease and decided to transfer the patient to a specialized cancer hospital.

Further pathological analyses of the specimen confirmed that the tumor was a primary vaginal melanoma. Immunocytochemistry results revealed that the tumoral cells were positive for HMB45, Melan-A, S-100, CD56, CD99, EMA, and negative for PAS. A malignant melanoma was therefore confirmed by the pathological diagnosis. One month after the local excision, the patient presented at Cancer Center of Sun Yat-Sen University (Guangzhou, China) for further therapy. A 3×2 cm pigmented mass was identified in the middle of the anterior vaginal wall. The urethra was normal and there was no grossly palpable lymph node. The patient underwent a Piver III radical hysterectomy, bilateral pelvic lymphadenectomy, left adnexectomy, right salpingectomy, total vaginectomy, total urethrectomy, cystostomy, and suspension of right ovary. Specimen observation following the surgical procedure indicated that the length of the removed vagina was 8 cm. Histopathological results of the specimen revealed the entire vaginal wall was involved, no tumor tissue was present in the surgical margins, and no lymphatic invasion was identified. Immunohistochemistry demonstrated positive staining for HMB-45 and S-100. Three weeks later, the patient presented with a serous-type of leakage from the perineum which was proven as urine by laboratory tests. The patient was subsequently diagnosed with a vesico-vaginal fistula. A surgical procedure to repair the vesico-vagina fistula was thus performed. The general condition of the patient rapidly deteriorated and two months following the surgery, a gynecological examination revealed a pelvic metastasis. A 6×5 cm mass was palpated under the left recta with involvement in the retrovaginal septum. In addition, a 4×5 cm mass on the right recta was found, which was extended onto the pelvic wall. An enlargement of the inguinal lymph node on the right side was also identified. Six months following the surgery, the patient had difficulties in deffacating, and a CT scan indicated the mass had covered the entire pelvic cavity. The patient was then lost to follow up.

## Discussion

Primary malignant melanoma of the vagina is a rare gynecological malignancy that is associated with high risk of recurrence, distant metastases and a short survival time (5). It predominantly occurs in postmenopausal women. The tumor is most frequently located in the distal third of the anterior vaginal wall. The presented case study focused on a 35-year-old female who was admitted to the Gynecological Ward of The First People’s Hospital of Foshan due to vaginal bleeding for 1 month. On gynecological examination, the vaginal malignant melanoma took the shape of a blue-black or black-brown mass, with plaques or ulceration. Vaginal malignant melanomas are commonly pigmented but occasionally may be devoid of pigment or may contain both pigmented and non-pigmented lesions in a zosteriform pattern. It may therefore be difficult to identify a polypoid carcinoma from a melanoma, especially in amelanotic melanomas without melanin pigment (6). Advanced melanoma of the vagina may be misdiagnosed by gynecologists due to a lack of awareness of the potential diagnosis (7). Gynecologists should therefore pay attention to suspicious lesions together with complaints of vaginal bleeding and a palpable mass on the vagina. A preoperative biopsy of the mass is an advisable method to improve tumor detection in patients with a primary malignant melanoma of the vagina. Immunohistochemical staining positive for vimentin, protein S-100, Melan A, and HMB-45 should also be used to confirm the diagnosis. In the present case, the pathological results indicated a malignant melanoma, and immunohistochemical analyses demonstrated HMB-45 and S-100-positive staining, which further confirmed the diagnosis.

Conservative wide local excision (8), radical surgery (9,10), radiotherapy (11,12), immunotherapy and chemotherapy are the currently regarded therapeutic options, which can be used individually or in combination, for primary vaginal melanoma. Despite these treatment options, the overall 5-year survival rate for women with primary vaginal melanoma is ~18% (13). Frumovitz *et al* (14) presented 37 women with clinical and radiographical stage I vaginal melanoma, and found a 5-year survival rate of 20%. Furthermore, locally advanced disease was identified in most patients at the time of first admission, which is not suitable for primary curative therapy. The prognosis of primary vaginal melanoma is very low, with <10% patients surviving for >5 years. There has also been no evidence of therapeutic improvement on survival time in recent years (15).

Tumor size has been considered in some reports, as one of the most important prognostic factors (11,16). Buchanan *et al* (8) showed in a meta-analysis that there is a significant difference in the average survival time between patients with tumor diameters of <3 cm and ≥3 (41 and 12 months, respectively, P=0.0024). Other potential prognostic factors including age, FIGO stage, tumor location, invasion depth, pigmentation, ulceration, histology, cell type, number of mitoses, vein invasion, type of the surgical procedure, adjuvant radiotherapy, and chemotherapy have also been investigated in terms of effects on survival time (17). Conversely, Petru *et al* (11) found that histological features, such as cell type, mitotic count, ulceration, vessel and lymphatic involvement, or amelanosis did not correlate with survival time, which is consistent with the findings reported by Liu *et al* (18). In addition, the primary therapy is important in the prognosis of the disease. The primary treatment should aim to completely resect the tumor from tumor-free surgical margins, and evaluate the related lymph nodes for tumor involvement. The surgical approaches, including wide local excision, total vaginectomy, or radical extirpation with en bloc removal of the involved pelvic organs, have been considered the most important potentially curative options, which could increase the chances of a longer survival time of the patient, as compared with those treated non-surgically (14). There has currently been no consensus concerning which procedure is the optimal approach for achieving disease-free survival or improving the overall survival rate. Skowronek and Rozsak (19) reported that aggressive surgical therapy was the preferred therapeutic procedure, whereas Räber *et al* (20) suggested that radical surgery achieved the best results. A report by Geisler *et al* (21) advised primary pelvic exenteration for vaginal melanoma >3 mm of invasion. A case study presented by Gökaslan (22) reported a radical surgery for a case with primary vaginal melanoma, however, the patient succumbed from distant metastases 16 months following the procedure. DeMatos *et al* (23) suggested that a radical surgical procedure would not improve survival rate as compared with a wide local resection, but would improve the quality of life of the patients. Frumovitz *et al* (14) found an overall increase in survival of only 5 months following pelvic exenteration, as compared with a wide local or radical excision or adjuvant radiation. The study argued that without an obvious improvement in survival, surgeons may choose to perform a wide excision and adjuvant radiation, instead of a more radical and morbid pelvic exenteration. Definitive recommendations could not be proposed due to the low number of patients (n=4) who had undergone a pelvic exenteration in their study (14). The dissection of lymph nodes that are clinically negative for melanoma of the vagina has additionally remained under debate. The lymphatics of the lower third of the vagina and the vulva drain primarily to the superficial and deep inguinal nodes or the deep pelvic lymph nodes (22). Miner *et al* (4) and Coleman *et al* (24) suggested that routine lymphatic dissection would not be achievable because of the low rate of lymph node metastasis. Therefore, sentinel lymph node mapping has gained in popularity. In 1999, Rodier *et al* (12) documented an evaluation of the sentinel node using a radiopharmaceutical-directed mapping technique for a case of vaginal melanoma. In 2002, Nakagawa *et al* (25) reported an evaluation of the sentinel node using a dye injection method for a case of malignant melanoma. In 2003, Siu *et al* (26) presented a laparoscopic ultrasonographic detection of metastatic pelvic lymph nodes for a case of vaginal melanoma. All three approaches have facilitated determining whether a patient should undergo lymph node removal, radiotherapy or chemotherapy.

Radiotherapy is of limited benefit in improving the survival of patients with vaginal melanoma. However, Petru *et a1* (11) reported that radiotherapy may be of value as an alternative to surgery or an adjunct modality in patients with lesions <3 cm in diameter. Similarly, Miner *et al* (4) suggested application of adjuvant radiotherapy following surgery or primarily for tumors that cannot be removed surgically. Frumovitz *et al* (14) supported the use of wide local or radical excision followed by adjuvant radiation therapy as a reasonable approach to treat vaginal melanoma. Similar results have been reported in head and neck mucosal melanomas (27). Overall, radiotherapy may be used as a rescue treatment to improve the prognosis of patients with vaginal melanomas. Currently, there have been only a few reports on the efficacy of chemotherapy in vaginal melanoma. The effect of chemotherapy as a primary or adjuvant therapy on patient survival has not been determined.

Traditional cytotoxic agents, including platinum compounds, dacarbazine and temozolomide, either alone or in combination, have been evaluated in the treatment of melanoma as being of limited or no benefit. Local chemotherapy has been previously trialed to downsize tumors and ensure local control (28). In a study from Harting *et al* (29), a partial response was observed in 36% of the 11 patients treated with systemic chemotherapy, and the median survival time was 10 months. Baloglu *et al* (17) reported administration of cisplatin and tremozolamide chemotherapy for six cycles following surgery of one patient. The patient was alive and disease-free 18 months following the diagnosis of the disease. It was concluded that the surgery and chemotherapy may be considered as the appropriate treatment for this disease. Application of high-dose interferon-α-2b may also be an effective neoadjuvant treatment in melanoma. Clinical trials have focused on molecular-targeting therapy using various multikinase inhibitors (including sorafenib) (30). Drugs targeting placenta-derived mesenchymal stem cells or c-kit are activated to high levels in melanoma cells, and have therefore gained increasing interest. Therefore, improvement of molecular targeting drugs as a neoadjuvant treatment for advanced malignant melanoma may improve prognosis for this disease.

In the present case study, the patient was hospitalized for the second time due to a local relapse without any clinically identified distant metastases. A radical surgery procedure was performed to improve the chance of survival, although there was a significant chance of surgical complication. Following the surgery, a vesico-vaginal fistula occurred. The fistula was repaired 3 weeks following the radical surgery, thus resulting in delayed adjuvant radiotherapy. The patient was lost to follow-up with pelvic metastases 7 months after the diagnosis of the disease. The result achieved in this case was not satisfactory in terms of a tumor-free period, even if a local eradication of the tumor was achieved to amend the primary operation. The experience and surgical skills of the surgical team is crucial in determining the type of surgery to be performed. Prior to the decision of the course of treatment for a vaginal tumor, the tumor should be biopsied to consider the malignant vaginal melanoma and the undetectable distant metastases. The surgeon should perform a comprehensive and careful consideration for the radical extirpation of potential complications. Overall, surgery together with adjuvant therapy should be considered an appropriate treatment for the disease.

Although primary vaginal melanoma is a rare malignant disease, attention should be given to suspicious pigmented lesions in routine gynecological examinations. A biopsy and evaluation of the pelvic nodes is important. The prognosis of vaginal melanoma remains poor, but early diagnosis and application of the appropriate combined modality therapy centered on the surgery, could improve the prognosis of this disease.
